# Close Homolog of L1 Deficiency Exacerbated Intestinal Epithelial Barrier Function in Mouse Model of Dextran Sulfate Sodium-Induced Colitis

**DOI:** 10.3389/fphys.2020.584508

**Published:** 2020-11-06

**Authors:** Ying Han, Xiaomeng Wang, Xiang Cheng, Ming Zhao, Tong Zhao, Liang Guo, Dan Liu, Kuiwu Wu, Ming Fan, Ming Shi, Lingling Zhu

**Affiliations:** ^1^Beijing Institute of Brain Disorders, Laboratory of Brain Disorders, Ministry of Science and Technology, Collaborative Innovation Center for Brain Disorders, Capital Medical University, Beijing, China; ^2^Institute of Military Cognition and Brain Sciences, Academy of Military Medical Sciences, Beijing, China; ^3^Jiangsu Center for the Collaboration and Innovation of Cancer Biotherapy, Cancer Institute, Xuzhou Medical University, Xuzhou, China; ^4^Co-innovation Center of Neuroregeneration, Nantong University, Nantong, China

**Keywords:** CHL1, dextran sulfate sodium (DSS), colitis, intestinal barrier, neutrophil, macrophage

## Abstract

The cell adhesion molecule CHL1, which belongs to the immunoglobulin superfamily, functions in a variety of physiological and pathological processes, including neural development, tissue injury, and repair. We previously found that the loss of CHL1 exacerbated the dextran sulfate sodium (DSS)-induced colitis in mice. In the present study, we further addressed the role of CHL1 in mouse model of DSS-induced colitis and its’ potential mechanism. Colon tissues were collected from CHL1^+/+^, CHL1^+/−^, and CHL1^−/−^ mice after DSS induction to investigate the effects of CHL1 on the development of colitis. The data showed that CHL1 was expressed in intestine tissue, and expression of CHL1 was increased by DSS-induced inflammation. CHL1 deficiency induced more pronounced colitis features, exacerbated inflammation, and damage to colonic tissues in DSS-induced mice. Moreover, colonic tissues of CHL1^−/−^ mice showed a marked increase in neutrophil and macrophage infiltration, be accompanied by more severe damage to intestinal epithelial cells and higher fluorescein isothiocyanate (FITC) leakage. Our results revealed deficiency of CHL1 exacerbated DSS-induced colitis, and this pathogenesis was potentially mediated by disruption of intestinal barrier integrity, indicating that CHL1 may be an attractive therapeutic target for inflammatory bowel diseases (IBDs) in mice.

## Introduction

Inflammatory bowel disease (IBD), which includes ulcerative colitis (UC) and Crohn’s disease (CD), is a chronic, idiopathic, relapsing disorder of the gastrointestinal tract (Xavier and Podolsky, 2007). UC is characterized by inflammation that is limited to the colon. In UC, the pattern of inflammation of the colonic mucosa includes impairment of the immune response, breakdown of the epithelial barrier, and enhancement of the inflammatory process. In contrast, CD involves any part of the gastrointestinal tract. The microscopic features of CD include a thickened submucosa, transmural inflammation, fissuring ulceration, and noncaseating granulomas (Liu and Stappenbeck, 2016).

Although the mechanisms of IBD remain unclear, this condition is viewed as the outcome of a multifactorial process, involving alterations in innate immunity and the immune response to bacteria, genetic predispositions, and some environmental factors (Gaya et al., 2006). Genome-wide association studies have identified several variants associated with IBD. There are now more than 200 IBD risk loci. It has been suggested that IBD is related to disorders of the innate immune response, adaptive immunity, endoplasmic reticulum stress, autophagy, intestinal epithelial barrier function, and microbial defense pathways (Liu et al., 2015). In patients with CD, the genes encoding adhesion molecules may lead to uncontrolled inflammation with ensuing destruction of epithelial cells, inappropriate stimulation of antimicrobial and T cell differentiation, and inflammasome events (Palmieri et al., 2015).

Adhesion molecules have been reported to regulate the recirculation of leukocytes. Leukocyte recruitment is pivotal for the initiation and perpetuation of IBD and is controlled by the specificity and interactions of chemokines and adhesion molecules (Springer, 1994). Interactions between the adhesion molecules a4b7-integrin and mucosal addressin cell-adhesion molecule-1 (MAdCAM-1) promote the accumulation of pathogenic T cell populations in the inflamed intestine (Schippers et al., 2016). Additionally, adhesion molecules maintain intestinal barrier function, which is crucial in preventing intestinal inflammation. A dual immunoglobulin domain-containing adhesion molecule (DICAM) has been recently identified and is known for its involvement in cell-cell adhesion through homophilic and heterophilic interactions with integrin αVβ3, which affects the severity of colonic inflammation (Jung et al., 2008).

**Figure 1:**
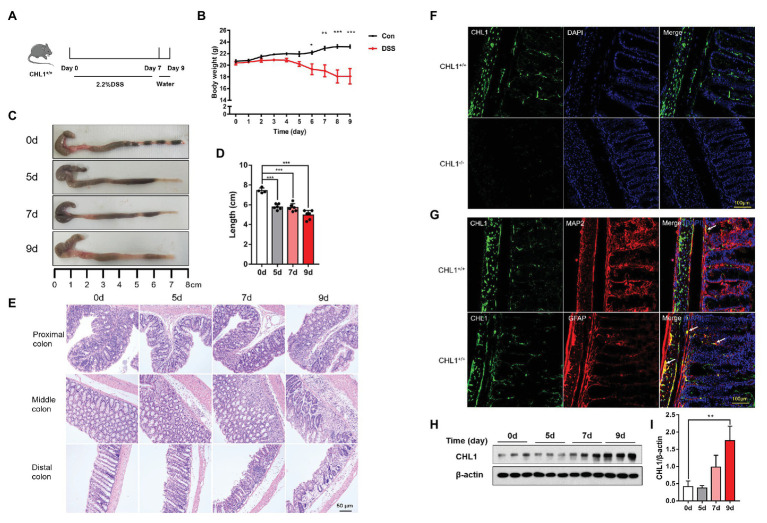
CHL1 expression was increased in dextran sulfate sodium (DSS)-induced colitis. **(A)** DSS-induced acute colitis mouse model. CHL1^+/+^ mice had *ad libitum* access to sterile drinking water containing 2.2% DSS for 7 days, followed by normal drinking water for 2 days. The mice were euthanized on the 5th, 7th, and 9th days after the initiation of DSS treatment. **(B)** Body weight loss in the DSS-induced acute colitis mouse model (^*^p < 0.05, ^**^p < 0.01, ^***^p < 0.001, *n* = 4–6/group). **(C,D)** Colon sections obtained from mice were analyzed for their outward appearance; time-dependent effects of DSS treatment on colon length (^***^p < 0.001; *n* = 4–6/group). **(E)** Time-dependent effects of DSS treatment on representative histological images of colonic tissue collected from CHL1^+/+^ mice. **(F)** CHL1 (green) expression was detected in the colonic tissue of CHL1^+/+^ mice, while CHL1 was totally negative in the colon from CHL1^−/−^ mice by using an immunofluorescence assay. **(G)** Representative immunofluorescent images indicate that CHL1 (green) is co-localized with microtubule-associated protein 2 (MAP2; red) and glial fibrillary acidic protein (GFAP; red) in mouse colonic sections. **(H,I)** Western blots **(H)** and analysis **(I)** showing the time-dependent effects of DSS treatment on CHL1 expression in the colon tissue of CHL1^+/+^ mice (^**^p < 0.01, *n* = 3/group).

The cell adhesion molecule CHL1, also known as L1CAM2, is a member of the immunoglobulin superfamily (IgSF). Previous reports have demonstrated that CHL1 mainly participates in multiple aspects of neural development and regeneration after injury (Zhang et al., 2000; Yamanaka et al., 2011). CHL1 is expressed not only in neurons but also in astrocytes and circulating leukocytes, which suggests that CHL1 has a variety of other important functions. The cell adhesion molecule L1 is highly homologous to CHL1, and blocking L1 inhibits T cell adhesion and attack of neurons *in vitro*. Downregulation of neuronal L1, which may involve the transcriptional repressor REST, is an adaptive attempt to promote neuronal self-defense in response to neuroinflammation (Menzel et al., 2016). Furthermore, astrogliosis stimulated by bacterial lipopolysaccharide (LPS) upregulates CHL1 expression in primary cultures of mouse cerebral astrocytes, coinciding with increased protein synthesis and translocation of protein kinase δ (PKCδ) from the cytosol to the membrane fraction (Wu et al., 2010). Previous research of our group showed that CHL1^−/−^ mice had more severe colitis features, such as weight losing, fecal blood, and shortening of colon length (Wang et al., 2018), but its mechanism and pathological effects were unclear. Our results showed colocalization of CHL1 with glial fibrillary acidic protein (GFAP)-positive glial cells in mouse colon tissue (Figure 1G). Here, we investigated the effects of CHL1 on the development of dextran sulfate sodium (DSS)-induced colitis.

To study the correlation between CHL1 and colitis, we used CHL1^+/+^, CHL1^+/−^, and CHL1^−/−^ mice in a DSS-induced colitis mouse model. We found that CHL1 expression was increased in the distal colon in a time-dependent manner after DSS treatment. CHL1 deficiency induced more pronounced colitis features in DSS-induced colitis mice than in wild-type, and CHL1^−/−^ mice showed a marked increase in neutrophil and macrophage infiltration into colonic tissues, cytokines, and other factors might together contribute to the injury of gut barrier function. This study provides a novel functional role of CHL1 in regulating colitis.

## Materials and Methods

### Mouse Models

In all experiments, the ethics guidelines for investigations of conscious animals were followed, and the experiments were approved by the Institutional Animal Care and Use Committee of Institute of Cognition and Brain Sciences (NO. IACUC-2017049). Wild-type mice were purchased from Laboratory Animal Center of Vital River Experimental Animal Company (Beijing, China). CHL1^−/−^ mice were previously described (Montag-Sallaz et al., 2002). The background of CHL1^−/−^ mice was the C57BL/6 strain. CHL1^+/−^ mice were established by breeding CHL1^−/−^ mice with C57BL/6 mice. All mice were maintained under specific-pathogen-free conditions under a 12 h light/dark cycle. Male mice aged 7–8 weeks were used in this study.

To explore the alternations in CHL1 levels in response to colitis, CHL1^+/+^ mice were fed sterile drinking water containing 2.2% DSS (MP Biomedicals, United States) for 7 days *ad libitum*, followed by normal drinking water for 2 days. Mice were euthanized on the 5th, 7th, and 9th days after the initiation of DSS treatment. Then, the colon was removed and washed with PBS for subsequent assays.

To explore the effects of CHL1 deficiency on the development of DSS-induced colitis, CHL1^+/+^, CHL1^+/−^, and CHL1^−/−^ mice were exposed to sterile water containing 1.5% DSS *ad libitum*, and the CHL1^−/−^ and CHL1^+/−^ mice were more sensitive to DSS-induced colitis than CHL1^+/+^ mice. The mice were euthanized on the 9th day after DSS exposure, and the colon was removed for subsequent assays.

### Immunofluorescence Staining

The mice were anesthetized with 1% sodium pentobarbital by intraperitoneal injection and were perfused with chilled 0.9% saline to flush out the circulating blood, followed by perfusion with 4% paraformaldehyde. Colon tissues were collected from CHL1^+/+^ and CHL1^−/−^ mice. After each colon was dehydrated and frozen-sectioned at a thickness of 9 μm, the sections were blocked with 5% BSA for 30 min at 37°C and then incubated with specific primary antibody (CHL1, 1:50, R&D, United States) overnight at 4°C, followed by incubation with secondary antibodies (Alexa Fluor 488, 1:500, Thermo, Waltham, MA) for 90 min at 37°C. Nuclei were counterstained with DAPI-containing mounting medium (ZSGB-BIO, CN). Images were captured using a scanning confocal microscope (Nikon, Tokyo, Japan).

### Western Blot Analysis

The lysates were prepared from mouse distal colon tissues, and the total protein concentration was measured. Each sample was loaded onto an SDS-PAGE gel and was separated by electrophoresis. Then, the proteins were transferred to nitrocellulose membranes. After the membranes were blocked, the specific primary antibodies (CHL1, 1:1,000 dilution, R&D; β-actin, 1:10,000 dilution, Sigma) were applied overnight at 4°C. The membranes were then washed and incubated with HRP-conjugated rabbit anti-goat (1:5,000 dilution; Bio-Rad, Hercules, CA) or goat anti-mouse secondary antibody (1:10,000 dilution; Bio-Rad) for 1 h at room temperature. The specific bands were visualized using an ECL detection kit (Bio-Rad).

### Quantitative Real-Time PCR

Total RNA was extracted from distal intestinal tissue homogenates using TRIzol reagent (Invitrogen, Carlsbad, CA). cDNA was synthesized by a reverse transcription kit (Vazyme Biotech Co.,Ltd, China) according to the manufacturer’s instructions. Real-time PCR was performed with SYBR Green master mix (Genstar Biotech, China) using a real-time PCR detection system as recommended by the manufacturer. The following oligonucleotide primers were used: β-actin: forward: 5'-ACTGTCGAGTCGCGTCCA-3', reverse: 5'-GTCATCCATGGCGAACTGGT-3'; interleukin-1β (IL-1β): forward: 5'-TTCAGGCAGGCAGTATCACTC-3', reverse: 5'-GAAGGTCCACGGGAAAGACAC-3'; interleukin-6 (IL-6): forward: 5'-AGTCCTTCCTACCCCAATTTCC-3', reverse: 5'-TTGGTTAGCCACTCCTTC-3'; and tumor necrosis factor alpha (TNF-α): forward: 5'-CCCTCACACTCAGATCATCTTCT-3', reverse: 5'-GCTACGACGTGGGCTACAG-3'. Gene-specific expression was normalized to β-actin expression.

### Assessment of Colitis Symptoms and Disease Activity Index

To explore the effects of CHL1 deficiency on the development of DSS-induced colitis, the animal body weight, stool consistency, and the presence of gross rectal blood were each evaluated daily. Each parameter was assigned a score according to previously described criteria (da Silva et al., 2016; Bibi et al., 2017) and used to calculate an average daily disease activity index (DAI; Supplementary Table 1).

### Histological Staining

The mice were perfused with saline to flush out circulating blood cells under anesthesia. Subsequently, the colon tissues were collected, and the lengths were measured in a relaxed position without stretching. Then, the colon tissues were fixed in 10% buffered formaldehyde and embedded in paraffin. The colon tissues were cut and stained with hematoxylin and eosin (HE). HE-stained tissue sections were scanned by a NanoZoomer-XR Scanner C12000 (Hamamatsu Inc., JP) or imaged on an Olympus BX51 (Olympus, JP) microscope using the Spot Insight image capture system CCD camera.

### Histological Scoring

To examine the effects of CHL1 deficiency on the development of DSS-induced colitis by histological scoring, over 100 fields were being photographed in each group. For each field, inflammatory cell infiltration and tissue damage were assessed in a double-blind trial. The data represent the percentage of fields scored as normal, mild, moderate, and severe in each group. Inflammatory cell infiltration score was evaluated as described below: (1) normal: inflammatory cells in lamina propria occasionally; (2) mild: inflammatory cells in lamina propria increased; (3) moderate: confluent inflammatory cells infiltrating into submucosa; and (4) severe: inflammatory cells transmural extension. Tissue damage score was evaluated as described below: (1) normal: no mucosal damage; (2) mild: punctuate mucosal erosions; (3) moderate: surface mucosal or local ulcer erosion; and (4) severe: wide-ranging mucosal damage and extension into deeper structures of the bowel wall.

### *In vivo* Intestinal Permeability Assay

Intestinal permeability was measured by determining the amount of FITC-dextran in the blood after oral administration as described previously (Xie et al., 2014). On the 5th day, the mice were gavaged with 0.6 mg/g body weight FITC-dextran (Sigma-Aldrich, UK) for 4 h, and blood samples were taken from the hepatic vein. The blood samples were first centrifuged (3,000 rpm, 4°C, 30 min), and serum was collected and added to a 96-well microplate. The concentration of FITC was measured by a Fluoroskan Ascent Fc (Thermo Scientific, United States) at an excitation wavelength of 480 nm and emission wavelength of 530 nm using serially diluted samples of the marker as a standard. Then, the colon tissues were cleaned with ice-cold PBS. The distribution of FITC-dextran in the sectioned colonic tissue was determined by a Nikon Ti-A1 inverted fluorescent microscope.

### Immunohistochemical Staining

Immunohistochemical staining was performed as previously described (Carrascal et al., 2018). The sections were dewaxed in xylene and gradually hydrated in a decreasing ethanol series ending in distilled water. Endogenous peroxidase activity was quenched using 3% hydrogen peroxide in distilled water, and then, the sections were washed in PBS. After antigen retrieval by boiling the slides in 1 mM EDTA buffer (pH 8.0) for 10 min, the sections were blocked with 5% BSA in PBS for 40 min at 37°C. Then, the sections were incubated with the anti-Ly6B.2 (1:200 dilution, AbD Serotec, UK) or anti-F4/80 (1:1,000 dilution, Servicebio, CN) antibody in PBS containing 5% BSA overnight at 4°C. The sections were washed with PBS and then incubated with horseradish peroxidase-conjugated secondary antibodies (ZSGB-BIO, CN). The color was developed by incubation with 3,3'-diaminobenzidine solution. The sections were then counterstained with hematoxylin, dehydrated, and mounted. Images were obtained on an Olympus BX51 microscope (Olympus, JP) using the Spot Insight image capture system CCD camera. Staining was assessed microscopically by two independent pathologists in a blinded manner.

### Statistical Analysis

All data are expressed as arithmetic mean ± SEM. All statistical analyses were performed using GraphPad Prism version 7.0. Null hypotheses were rejected at *p* ≥ 0.05. For statistical comparisons between two groups, we first performed a Shapiro-Wilk normality test (prism) to determine whether the data were likely normally distributed. For normally distributed data, we used unpaired Student’s *t*-tests to evaluate the statistical significance of differences between the two groups. Statistically significant differences between groups were determined using one-way ANOVA followed by Dunnett’s tests. Two-way ANOVA followed by Bonferroni’s *post hoc* tests for multiple comparisons were used. For all analyses, *p* ≤ 0.05 was considered statistically significant.

## Results

### CHL1 Expression Was Increased in DSS-Induced Inflammatory Colitis

The DSS-induced colitis mouse model was used in the present study (Ruiz et al., 2016). To validate the DSS-induced acute colitis model, wild-type mice had *ad libitum* access to sterile drinking water containing 2.2% DSS for 7 days, followed by normal drinking water for 2 days (Figure 1A). DSS-treated mice exhibited colitis symptoms, as evidenced by a significant shortening of the colon (Figures 1C,D) and weight loss (Figure 1B). HE staining showed apparent inflammation in the DSS-treated groups, including extensive ulceration of the epithelial layer, edema, crypt damage of the bowel wall, and leukocyte infiltration into the mucosa (Figure 1E).

The expression pattern and localization of CHL1 in the colon tissue have not been previously reported. In the present study, immunofluorescence assays were used to detect CHL1 expression in the colon tissue of mice. Positive CHL1 staining was observed in the muscle layers of the colon tissue in CHL1^+/+^ mice. Positive CHL1 staining also showed small bundles, which sporadically localized in the submucosa. Moreover, positive CHL1 staining surrounded the large intestinal gland in a reticular manner (Figure 1F; Supplementary Figure 2A). To confirm the expression and specificity of CHL1 staining, we used CHL1^−/−^ mouse colon tissue. CHL1 staining was completely negative in the colon tissue of CHL1^−/−^ mice (Figure 1F). Colonic sections of CHL1^+/+^ mouse were stained to visualize CHL1 (green), microtubule-associated protein 2 (MAP2; red), and GFAP (red), as indicated to characterize CHL1 expression in mouse neuron and enteric glia (Figure 1G). We then detected the expression level of CHL1 protein in colon tissue in response to DSS-induced colitis by western blot assays. DSS treatment evidently upregulated CHL1 expression levels in mouse colon tissue on the 9th day (Figures 1H,I; Supplementary Figure 2B). These data suggest that CHL1 is related to the occurrence of colitis.

### CHL1 Deficiency Augmented DSS-Induced Colitis in Mice

**Figure 2:**
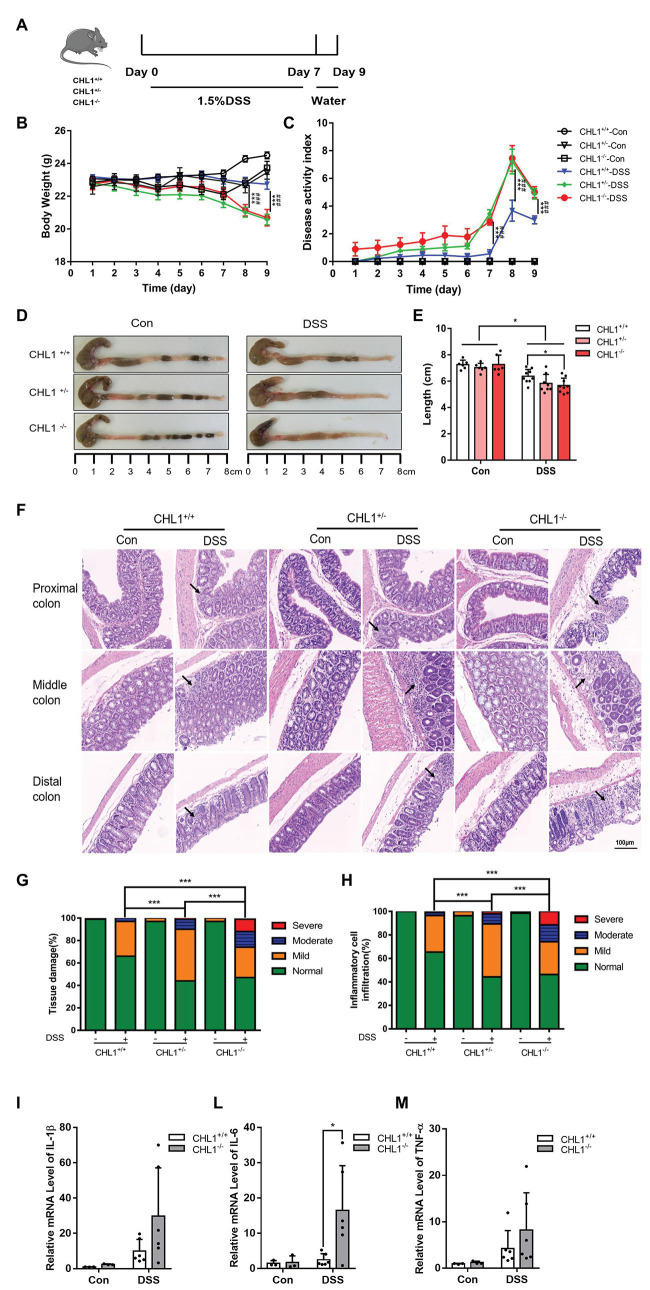
(Continued)FIGURE 2CHL1 deficiency augmented DSS-induced colitis in mice. **(A)** DSS-induced acute colitis mouse model. CHL1^+/+^, CHL1^+/−^, and CHL1^−/−^ mice had *ad libitum* access to sterile drinking water containing 1.5% DSS for 7 days, followed by normal drinking water for 2 days. The mice were euthanized on the 9th day after the initiation of DSS treatment. **(B,C)** Body weight loss and the disease activity index score in DSS-exposed CHL1^+/+^, CHL1^+/−^, and CHL1^−/−^ mice (DSS-induced CHL1^+/+^ group vs. DSS-induced CHL1^+/−^ group, ^***^p < 0.001, *n* = 6–9/group; DSS-induced CHL1^+/+^ group vs. DSS-induced CHL1^−/−^ group, ^###^*p* < 0.001, *n* = 6–9/group). **(D,E)** Colon sections obtained from mice were analyzed for their outward appearance; colon length in DSS-exposed CHL1^+/+^, CHL1^+/−^, and CHL1^−/−^ mice (^*^p < 0.05, *n* = 6–9/group). **(F)** Representative histological images of colonic tissue obtained from CHL1^+/+^, CHL1^+/−^, and CHL1^−/−^ mice with and without DSS treatment (the arrows indicate colon tissue injury). **(G,H)** The histology score of tissue damage and inflammatory cell infiltration in CHL1^+/+^, CHL1^+/−^, and CHL1^−/−^ mice with and without DSS treatment (^***^p < 0.001, *n* = 6–9/group). **(I,L,M)** Real-time PCR analysis of changes in the inflammatory cytokines interleukin-1β (IL-1β), interleukin-6 (IL-6), and tumor necrosis factor alpha (TNF-α) in the distal colon of mice. The increase in IL-6 expression in CHL1^−/−^ mice after DSS treatment was significant (^*^p < 0.05, *n* = 4–6/group).

According to literature reports for colitis mouse model induced by DSS, the dose of DSS ranges from 1 to 7% (Reber et al., 2006; Van Crombruggen et al., 2008). DSS concentration of 2.5% (w/v) in the drinking water for 7 days induces strong colitis, but low mortality rates (Deng et al., 2016; Wei et al., 2018). Therefore, in our preliminary experiment, 2.5% DSS had been used to induce colitis, but the CHL1^−/−^ mice were unable to tolerate and nearly half of the mice died on the 6th day (shown in Supplementary Figure 1). To further assess the impact of CHL1 on the development of DSS-induced colitis, CHL1^+/+^, CHL1^+/−^, and CHL1^−/−^ mice were given drinking water containing 1.5% DSS *ad libitum* (Figure 2A). At baseline and throughout the course of treatment, we measured the features of colitis on a daily basis. DSS-induced injury reproduces some clinical features of human colitis, including weight loss, diarrhea, and bloody stool. All control mice were negative for weight loss, diarrhea, and fecal blood. As expected, the body weights in CHL1^+/+^, CHL1^+/−^, and CHL1^−/−^ mice were decreased after DSS treatment. The body weight of CHL1^+/+^ mice was significantly higher than that of CHL1^+/−^ and CHL1^−/−^ mice on the 8th and 9th days after DSS treatment (Figure 2B). The DAI scores of CHL1^−/−^ and CHL1^+/−^ mice were significantly higher than those of CHL1^+/+^ mice on days 7, 8, and 9 after DSS induction (Figure 2C). These findings demonstrated that CHL1 deficiency exacerbated DSS-induced colitis and suggested a critical role of CHL1 in IBD.

Moreover, we observed that the length of the colon tissue in DSS-induced CHL1^−/−^ mice was significantly shorter than that in CHL1^+/+^ mice (Figures 2D,E). Importantly, HE staining revealed more severe colitis symptoms in DSS-induced CHL1^+/−^ mice than in DSS-induced CHL1^+/+^ mice. Furthermore, compared with the colon tissue of CHL1^+/−^ and CHL1^+/+^ mice, the colon tissue of CHL1^−/−^ mice exhibited severe inflammation and crypt damage (Figure 2F). The pathological changes of colonic tissue, including inflammatory cell infiltration and tissue damage, were scored. The histology scores in the DSS group mice were increased. DSS-induced CHL1^+/−^ mice had a significantly higher inflammatory score and tissue damage than DSS-treated CHL1^+/+^ mice. Moreover, the colon tissue of the CHL1^−/−^ group had a significantly higher inflammatory score and damage than the colon tissue of the CHL1^+/−^ and CHL1^+/+^ groups after DSS treatment (Figures 2G,H). However, all control mice were negative for inflammatory cell infiltration and tissue damage in colon tissue. The inflammatory cytokines IL-1β, IL-6, and TNF-α in the distal colon tissue were then measured by using real-time PCR. The data showed that the level of IL-6 in CHL1^−/−^ mice after DSS treatment was increased significantly, while the levels of IL-1β and TNF-α showed an increasing trend (Figures 2I,L,M). These results fully indicated that CHL1 deficiency exacerbated the development of DSS-induced colitis.

### CHL1 Deficiency Exacerbated Epithelial Barrier Function in Mice

**Figure 3:**
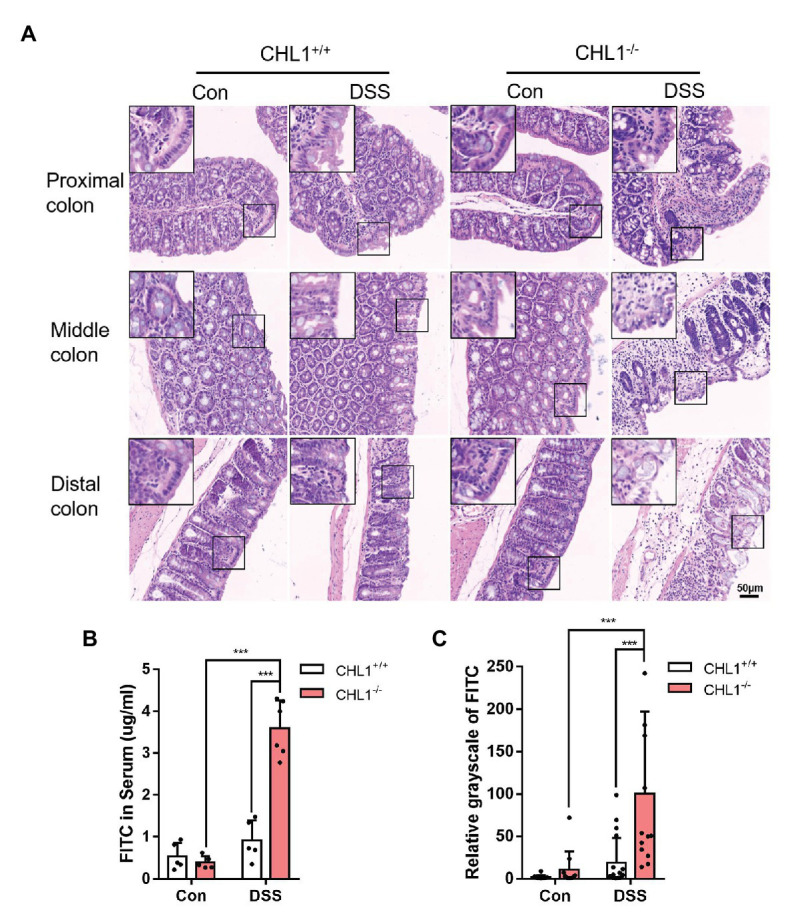
CHL1 deficiency exacerbated epithelial barrier damage in inflammatory bowel disease (IBD). **(A)** High magnification histological images of colonic tissue from CHL1^+/+^ and CHL1^−/−^ mice with and without DSS treatment showed altered intestinal epithelial cellular shape in tissues from CHL1^−/−^ animals. Insets show examples of areas of the epithelium under ultrahigh magnification. **(B)** Intestinal permeability was measured by the appearance of orally administered FITC-labeled dextran in the serum of DSS-exposed CHL1^+/+^ and CHL1^−/−^ mice (^***^p < 0.001, *n* = 5/group). **(C)** The relative grayscale values of fluorescence microscopy images of the intestinal mucosa from DSS-treated CHL1^+/+^ and CHL1^−/−^ mice exposed to orally administered FITC-labeled dextran (^***^p < 0.001, *n* = 5/group).

The previous results showed that CHL1^−/−^ mice had more severe colitis and more significant phenotypes than CHL1^+/−^ and CHL1^+/+^mice. Intestinal epithelial cell density plays a key role in epithelial barrier function (Yu et al., 2015). To further examine the effects of CHL1 deficiency, the intestinal epithelial barrier and intestinal barrier function were measured in CHL1^+/+^ and CHL1^−/−^ mice after DSS treatment. We performed high magnification microscopic analysis of the mucosal structure in CHL1^+/+^ and CHL1^−/−^ mice after DSS induction. Decreased intestinal epithelial cell density was observed after DSS treatment. Compared to CHL1^+/+^ mice, CHL1^−/−^ mice displayed lower epithelial density in colonic tissues, more disorganized columnar epithelial cells and increased cell volumes (Figure 3A). To evaluate intestinal barrier function, mice were administered an oral dose of FITC-dextran on the 5th day after DSS exposure, and the FITC level in the serum was measured 4 h later to determine intestinal permeability. DSS exposure induced a slight increase in intestinal permeability, as reflected by the increased level of FITC in the serum in CHL1^+/+^ mice, while the FITC level was markedly increased in CHL1^−/−^ mice (Figure 3B). The distribution of FITC-dextran in sectioned colonic tissue was evaluated by fluorescence microscopy. The results showed retention of FITC at the barrier in CHL1^+/+^ mice. However, FITC crossed the epithelial barrier after DSS exposure in CHL1^−/−^ mice. The changes in the relative intensity of FITC suggest that CHL1 deficiency impaired intestinal barrier function in colitis mice (Figure 3C). These data suggest that the detrimental effects of CHL1 deficiency could be due to decreased epithelial barrier function as a result of disordered intestinal epithelial cell arrangement and density.

### CHL1 Deficiency Induced Inflammatory Cell Infiltration

**Figure 4:**
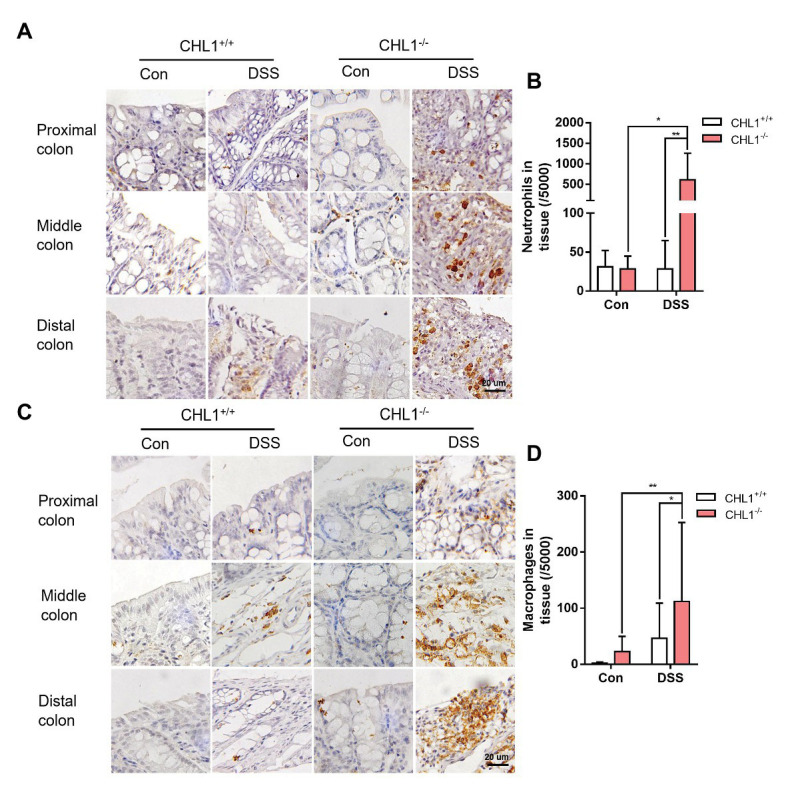
CHL1 deficiency induced inflammatory cell infiltration **(A,B)** Representative immunohistochemical images **(A)** and analysis **(B)** of Ly6B.2-stained colon tissue from DSS-exposed CHL1^+/+^ and CHL1^−/−^ mice (^*^p < 0.05, ^**^p < 0.01, *n* = 5/group). **(C,D)** Representative immunohistochemical images **(C)** and analysis **(D)** showing F4/80 staining in CHL1^+/+^ and CHL1^−/−^ mice exposed to DSS (^*^p < 0.05, ^**^p < 0.01, *n* = 5/group).

Neutrophils are key inflammatory cells in the innate defense against invading pathogens. The egress and recruitment of neutrophils to the site of inflammation are tightly controlled in physiological and pathological conditions. Excessive neutrophil infiltration contributes to tissue damage in inflammatory disorders (Kolaczkowska and Kubes, 2013). It has been proposed that neutrophil recruitment/infiltration is directly related to increased pathological damage in experimental colitis, although their relative contributions to the pathogenesis of IBD are still controversial (Davies and Abreu, 2015). Immunohistochemical staining with an antibody against Ly6B as a marker of neutrophils showed that neutrophil infiltration in the colon tissue of CHL1^−/−^ mice was significantly higher than that in CHL1^+/+^ mice after DSS treatment (Figures 4A,B), which suggested that CHL1 was probably related to changes in neutrophil infiltration.

Macrophages are an important element of the innate immune system and exhibit high heterogeneity in the two subtypes. The number of macrophages is always increased by the enhanced inflammation in IBD (Kmieć et al., 2017). To explore the effect of CHL1 deficiency on macrophage infiltration in DSS-induced mice, colon tissue was examined by immunohistochemical staining with an antibody against F4/80 as a marker of macrophages. Macrophage infiltration in CHL1^−/−^ mice was significantly higher than that in CHL1^+/+^ mice in the presence of DSS-induced colitis (Figures 4C,D). The data suggest that the changes in neutrophil and macrophage infiltration likely involved CHL1 deficiency.

## Discussion

**Figure 5:**
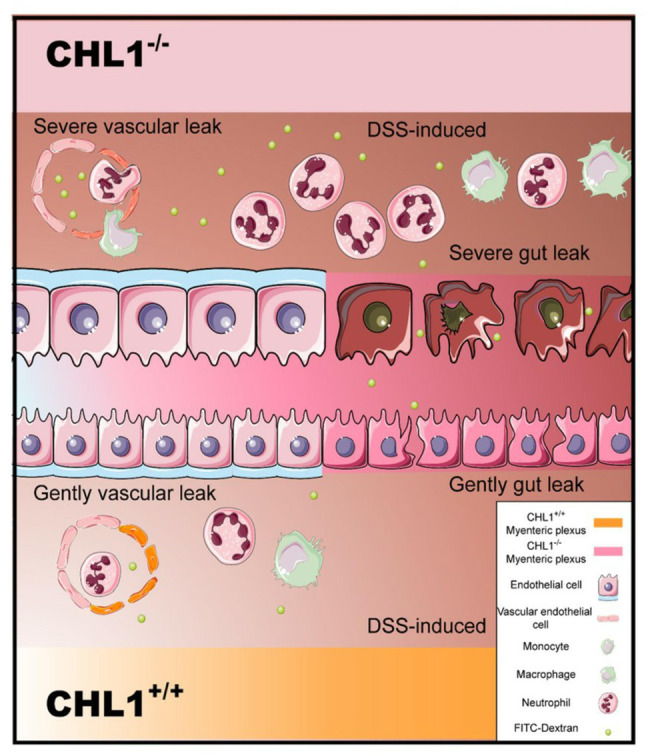
Model of CHL1 deficiency-mediated exacerbation in mouse colitis. The loss of CHL1 exacerbates DSS-induced intestinal inflammation and immune responses in mice. Lamina propria is edematous. Inflammation cells, such as neutrophils and macrophages, infiltrate epithelial layer cell. CHL1 deficiency damages intestinal epithelial cells, and leads to the injury of epithelial barrier function. Furthermore, FITC cross the epithelial barrier after DSS exposure in CHL1^−/−^ mice.

Here, we provide new information regarding the impact of CHL1 on DSS-induced colitis. The data showed that CHL1 expression was increased in the distal colon in a time-dependent manner in DSS-induced colitis. CHL1 deficiency exacerbated the development of DSS-induced colitis with pronounced colitis features, including an increase in proinflammatory cytokines and the leakage of FITC from the colon to the serum. These results suggested that CHL1 could be involved in regulating the occurrence and development of IBD (Figure 5).

According to the literature, CHL1 has a variety of functions under different pathological and physiological conditions, including aggravating and alleviating injuries. Glial scar expression of CHL1 limits recovery after spinal cord injury CHL1 is a glial scar component that restricts posttraumatic axonal growth and remodeling of spinal circuits by homophilic binding mechanisms. Our previous study had found that CHL1^−/−^ mice showed a dramatically lower mortality rate and an augmented ventilatory response after they were subjected to high altitude hypoxia (Huang et al., 2013). The results indicated that CHL1 plays multitudinous roles in a variety of pathological models. Combined the previous results about the function of CHL1, the recent publication of a paper found that CHL1-deficient reduces the inflammatory response in 4% DSS-induced mice colitis, by regulating the balance of Th17/Treg in mice with colitis (Yao et al., 2020). In the present study, CHL1 may plays an anti-inflammation role initially in DSS-induced colitis, the increased of CHL1 attempts to reduce inflammation in WT mice colon. However, the current results cannot exclude this possibility that CHL1 have dual roles by mediating colitis in different DSS doses. More works need to be done to confirm in the future.

The intestinal epithelium can be easily disrupted during gut inflammation, as observed in IBD (Gitter et al., 2001). The intestinal epithelium might play a major role in the development and perpetuation of IBD (Wyatt et al., 1993). In the present study, during initial DSS administration, intestinal epithelial functions were impaired and substantially preceded the development of colitis in CHL1-deficient mice. Moreover, the distribution of FITC-dextran in sectioned colonic tissue was evaluated after CHL1^−/−^ mice were administered DSS. However, we found that CHL1 is not expressed in the intestinal epithelium (data not shown), and some positive staining of CHL1 co-localized with MAP2 (Figure 2G). Interestingly, CHL1 is predominantly expressed in enteric glia (Figure 2G). CHL1 plays a role in regulation of neuronal differentiation and survival, neurite outgrowth, and axon guidance (Maness and Schachner, 2007). There is no report on whether MAP2 levels are altered after CHL1 deletion. However, it is well-known that enteric glia activation has been reported to amplify intestinal inflammation (Grubišić and Gulbransen, 2017). Then, we have previously detected the expression of GFAP in CHL1 deficiency, and colon tissues were collected from CHL1^+/+^and CHL1^−/−^ mice. The data showed that the expression of GFAP was increased by CHL1 deficiency (Supplementary Figure 3).Enteric glia are distributed throughout the laminar structure of the gastrointestinal tract and closely appose neurons, immune cells, blood vessels, and the intestinal epithelium (Fu et al., 2013). Mucosal glia in the lamina propria directly underlie the epithelium, and this close proximity raised the possibility that enteric glia plays a role in regulating epithelial functions, such as cellular proliferation and barrier maintenance. When the GFAP promoter was used to express cellular toxins that eliminate glia in mice, intestinal epithelial permeability and proliferation increased, leading to the translocation of luminal bacteria and intestinal inflammation (Bush et al., 2001). Therefore, consistent with this finding, CHL1 deficiency in enteric glia might damage colonic tissues and increase the leakage of FITC from the colon to the serum. This damage resulted in the development of DSS-induced colitis. We hypothesized that the function of CHL1 might be mediated by glial cells. The specific mechanism and whether CHL1 is related to other cells need further study. It has been reported that L1 is expressed in T cells, and blocking L1 inhibits T cell adhesion (Menzel et al., 2016). However, it is still unclear whether CHL1 regulates the function of T cells.

In addition, adhesion molecules have been reported to maintain intestinal barrier function, which is crucial in preventing intestinal inflammation (Han et al., 2019). Inflammatory cytokine expression is associated with the severity of IBD. Excessive proinflammatory cytokines can damage the colonic mucosa and affect intestinal homeostasis. Proinflammatory cytokines, including IL-1β, IL-6, and TNF-α, have been implicated in the pathophysiology of IBD (Neurath, 2014; Chi et al., 2018). It has been reported that the extent of neutrophil infiltration is correlated with severity of DSS-induced intestinal inflammation (Larmonier et al., 2011). As Deng et al. (2016) described, the numbers of infiltrated neutrophils in the colon tissues were significantly higher in the DSS-induced mice with chronic unpredictable stress than WT mice, correlated with severity of disease. Moreover, the time-dependent effect on cellular infiltration in the DSS-induced colon tissue was observed, with progressive accumulation of macrophages F4/80^+^, T helper CD4^+^ (Th), T cytotoxic CD8^+^ (Tcyt) and T regulatory CD25^+^ (Treg) cells, and progressive changes in colonic pathology including destruction of crypts, loss of goblet cells, and depletion of the epithelial barrier (Nunes et al., 2018). These results suggest a relationship of neutrophil infiltration and severity of colitis. Under the same DSS treatment, the severity of colitis was different in CHL1^−/−^ mice, and the numbers of infiltrated neutrophils in the colon tissues were variable. May be due to the large individual differences in the neutrophil infiltration, neutrophil data seem to fall into two distinct groups (Figure 4).

We examined whether the change in CHL1 expression affected colonic inflammation and found that CHL1 expression was increased in a time-dependent manner during the development of DSS-induced colitis. In the present study, it is possible that CHL1 may play an anti-inflammation role in DSS-induced colitis. The data show that the level of IL-6 in CHL1^−/−^ mice after DSS treatment was increased significantly, while the levels of IL-1β and TNF-α showed an increasing trend (Figures 2I,L,M). Thus, the increased expression of CHL1 is involved in anti-inflammation in DSS-induced colitis for WT mice, while this ability of reduce colitis has been lost in CHL1^−/−^ mice. Accordingly, we speculate that deficiency of CHL1 exacerbates the development of colitis. Consistent with this finding, CHL1 expression increased in response to LPS-induced brain astrocyte activation through NF-κB signaling (Wu et al., 2010). However, in contrast, in the present study, IL-6 expression, as well as the number of neutrophils and macrophages, was significantly increased in colonic tissue in the CHL1^−/−^ group after DSS administration. This effect may be related to different regulatory mechanisms in the gut and brain tissue. Therefore, the precise mechanism by which CHL1 deficiency regulates inflammation during colitis needs to be addressed in the future.

Recently, a new role for CHL1 outside the nervous system has emerged. It was found that CHL1 is a significant factor during the malignant progression of cancer. However, the functional roles of CHL1 in physiological and pathological processes are poorly understood. Downregulation of CHL1 was detected in several types of tumors (such as stomach, rectal, colon, small intestinal, pancreatic, kidney, bladder, breast, thyroid, vulvar, and skin cancer), suggesting that CHL1 might act as a putative tumor suppressor (Senchenko et al., 2011). Interestingly, the CHL1 gene is hypermethylated in DNA samples from African American patients with colorectal carcinoma. It has been known that patients with IBD are at increased risk for the development of colorectal cancer compared to the general population (Lasry et al., 2016) and the same alleles overlapped in colorectal cancer and IBD in a mouse model (Kraak, 2015; Johnson et al., 2016). IBD, which is mediated by chronic intestinal inflammation, is widely accepted as one of the main risk factors leading to colorectal cancer (Kim and Chang, 2014). Whether CHL1 plays a potential role in colon cancer development through its regulation of the inflammatory processes of the intestine remains to be elucidated.

This work demonstrated that CHL1 deficiency exacerbated the occurrence and development of DSS-induced colitis with pronounced colitis features, including impaired epithelial barrier function and enhanced inflammation and damage to colonic tissues in mice with DSS-induced colitis. These results suggested that CHL1 could be involved in regulating the occurrence and development of colitis, which provides a novel functional role of CHL1 in regulating IBD. So far, no literature has reported that the change of CHL1 expression on IBD patients. Herein, we speculate that CHL1 might be an important biomarker for the diagnosis of IBD patients, and this study has a certain significance in clinic.

## Data Availability Statement

The raw data supporting the conclusions of this article will be made available by the authors, without undue reservation.

## Ethics Statement

The animal study was reviewed and approved by the Institutional Animal Care and Use Committee of Institute of Cognition and Brain Sciences (NO. IACUC-2017049).

## Author Contributions

XW, YH, MS, and LL conceptualized the study. YH and XW prepared and maintained CHL1+/+, CHL1+/−, and CHL1−/− mice, designed and performed morphological analysis and biochemical assays, and wrote the manuscript. MZ, KW, and XC supervised the project. All authors contributed to the article and approved the submitted version.

### Conflict of Interest

The authors declare that the research was conducted in the absence of any commercial or financial relationships that could be construed as a potential conflict of interest.

## Supplementary Material

Supplementary Figure 1 The survival rate of mice after treatment with 2.5% DSS for 7 days. Mice were treated with 2.5% DSS for 7 days, the CHL1^−/−^ mice were unable to tolerate and nearly half of the mice died on the 6th day (*n* = 5/group).

Supplementary Figure 2 CHL1 expression was increased in DSS-induced colitis. **(A)** CHL1 (green) expression was detected in the colonic tissue (proximal, middle, and distal) of CHL1^+/+^ mice. **(B)** Immunofluorescence showing the effects of DSS treatment on CHL1 expression in the colon tissue of CHL1^+/+^ mice, the upregulation of CHL1 located in muscularis and mucosa of colon (see arrow; *n* = 3/group).

Supplementary Figure 3 Glial fibrillary acidic protein (GFAP) levels were increased in mouse colon by CHL1 deficiency. **(A,B)** Colon tissues were collected from CHL1^+/+^ and CHL1^−/−^ mice, Western blots **(A)** and analysis **(B)** showed that the colon GFAP protein levels were increased in CHL1^−/−^ mice (^**^*p* < 0.01, *n* = 3/group).

## Abbreviations

DSS: Dextran sulfate sodium
IBD: Inflammatory bowel diseases
UC: Ulcerative colitis
CD: Crohn’s disease
FITC: Fluorescein isothiocyanate
HE: Hematoxylin and eosin
IL-1β: Interleukin-1β
IL-6: Interleukin-6
TNF-α: Tumor necrosis factor alpha
MAdCAM-1: Mucosal addressin cell-adhesion molecule-1
DICAM: Dual immunoglobulin domain-containing adhesion molecule
IgSF: Immunoglobulin superfamily
LPS: Lipopolysaccharide
PKCδ: Protein kinaseδ
GFAP: Glial fibrillary acidic protein
DAI: Disease activity index
MAP2: Microtubule-associated protein 2
