# Supplementary figures and images for: Close Homolog of L1 Deficiency Exacerbated Intestinal Epithelial Barrier Function in Mouse Model of Dextran Sulfate Sodium-Induced Colitis
Source: Front Physiol. 2020 Nov 6;11:584508. doi: 10.3389/fphys.2020.584508 (PMC7677258; doi:10.3389/fphys.2020.584508)

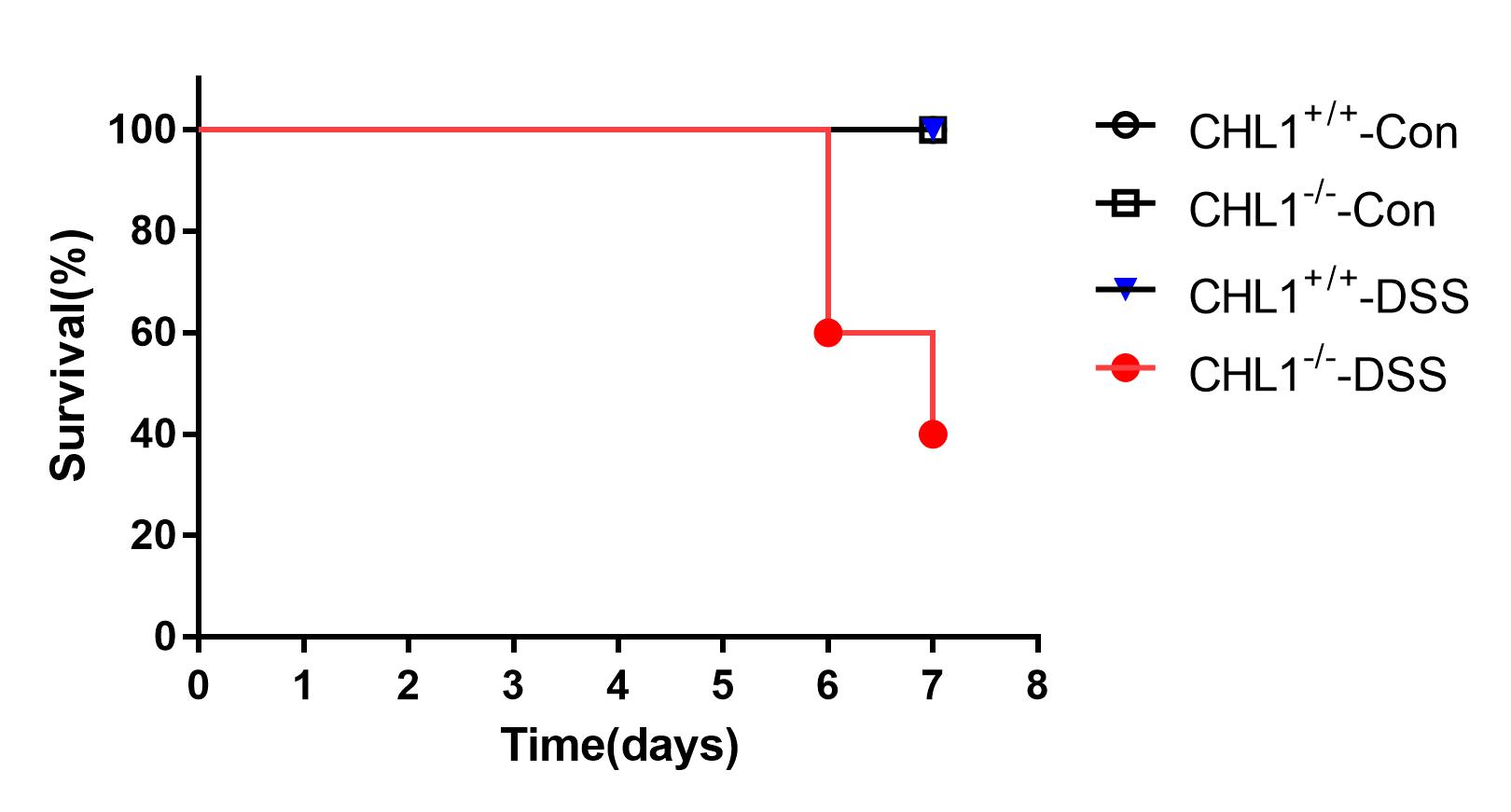

Supplement: Supplementary Figure 1 — The survival rate of mice after treatment with 2.5% DSS for 7 days. Mice were treated with 2.5% DSS for 7 days, the CHL1−/− mice were unable to tolerate and nearly half of the mice died on the 6th day (n = 5/group). [file Data_Sheet_1.zip › figures/Sfigure1.jpg]

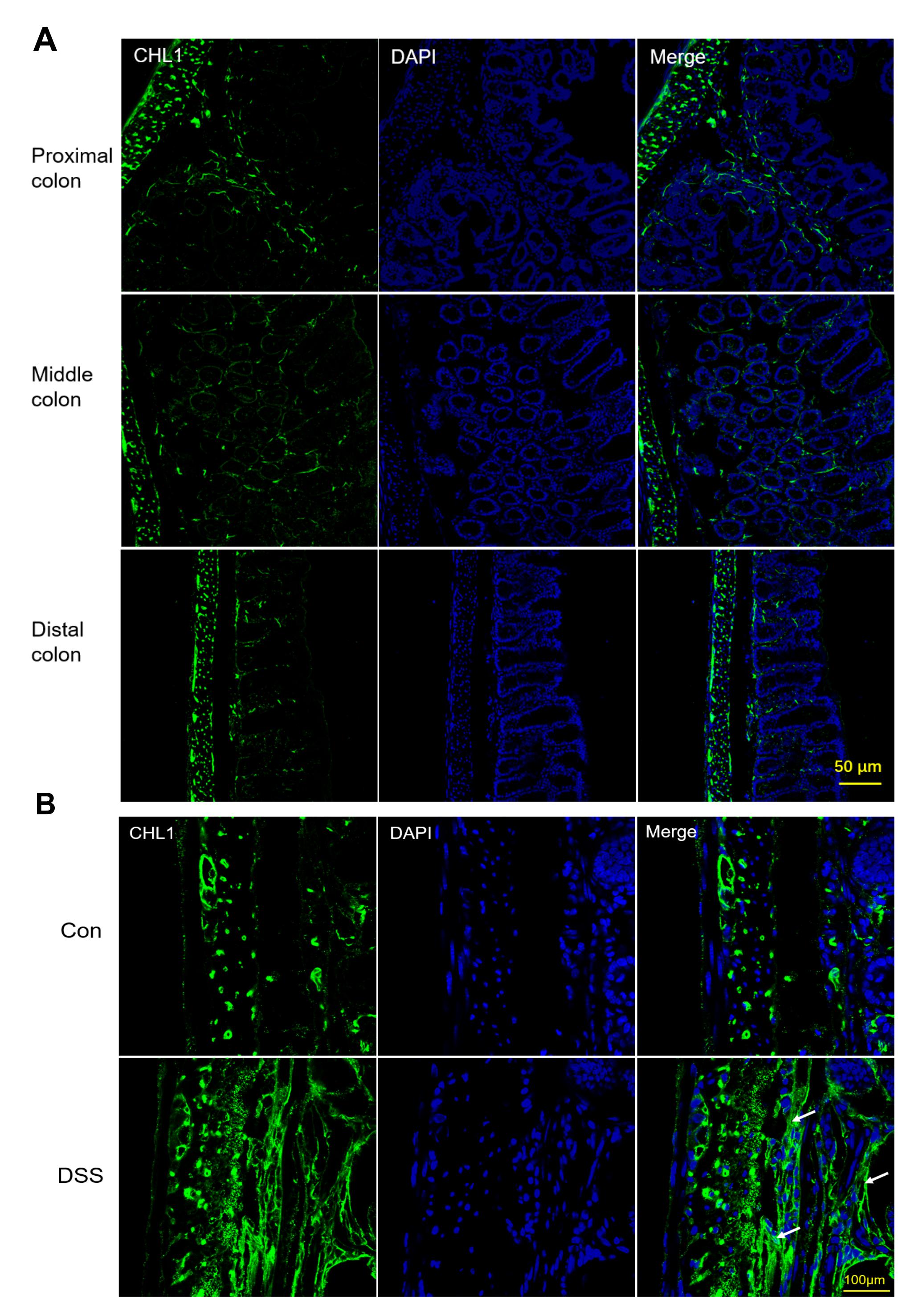

Supplement: Supplementary Figure 1 — The survival rate of mice after treatment with 2.5% DSS for 7 days. Mice were treated with 2.5% DSS for 7 days, the CHL1−/− mice were unable to tolerate and nearly half of the mice died on the 6th day (n = 5/group). [file Data_Sheet_1.zip › figures/Sfigure2.jpg]

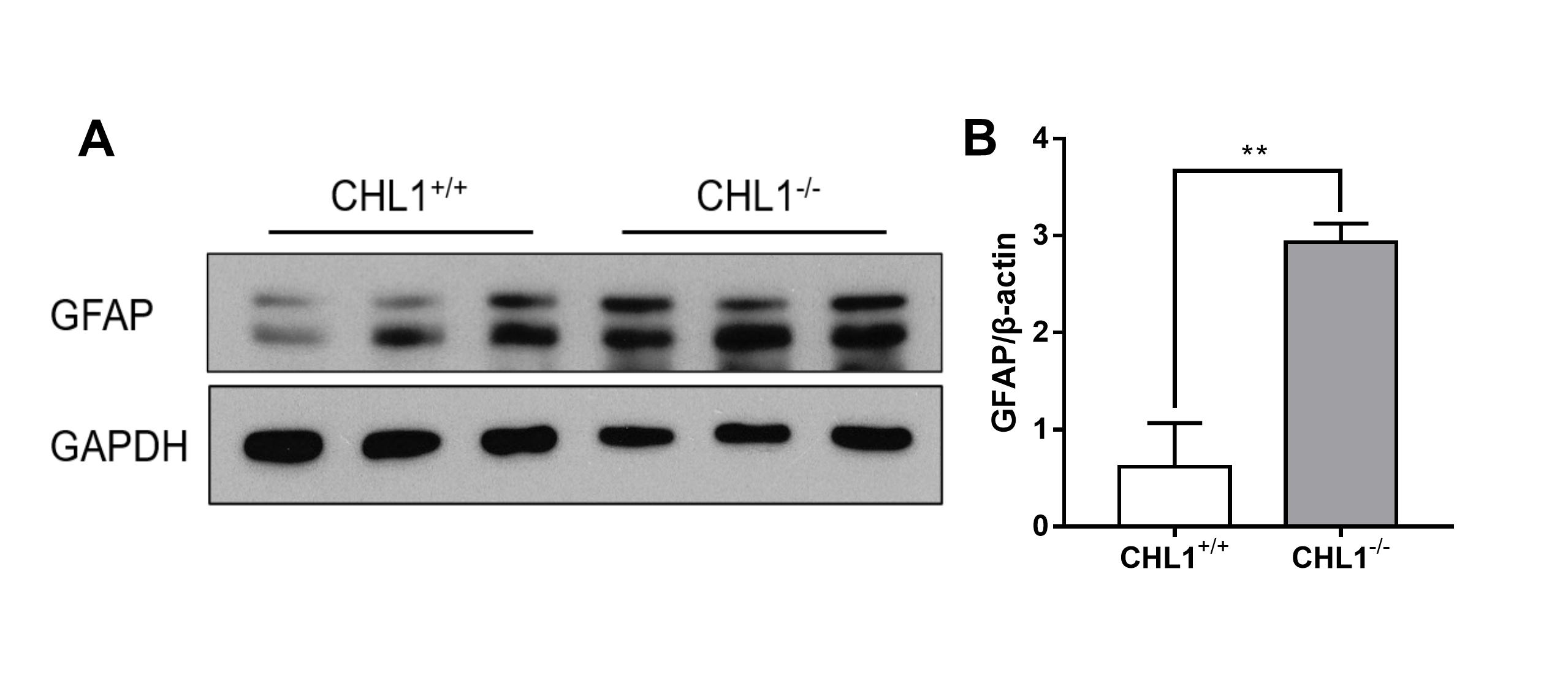

Supplement: Supplementary Figure 1 — The survival rate of mice after treatment with 2.5% DSS for 7 days. Mice were treated with 2.5% DSS for 7 days, the CHL1−/− mice were unable to tolerate and nearly half of the mice died on the 6th day (n = 5/group). [file Data_Sheet_1.zip › figures/Sfigure3.jpg]
