# Supplementary material for: Close Homolog of L1 Deficiency Exacerbated Intestinal Epithelial Barrier Function in Mouse Model of Dextran Sulfate Sodium-Induced Colitis
Source: Front Physiol. 2020 Nov 6;11:584508. doi: 10.3389/fphys.2020.584508 (PMC7677258; doi:10.3389/fphys.2020.584508)
Supplement: Supplementary file 2 [file Table_1.doc]

**Supplementary Table 1. DAI score**

| Score | Weight loss | Stool consistency | Rectal bleeding |
| --- | --- | --- | --- |
| 0 | None | Normal | Normal |
| 1 | 1-5% | - | - |
| 2 | 5-10% | Loose stools | - |
| 3 | 10-20% | - | - |
| 4 | >20% | Diarrhea | Gross bleeding |

DAI value is composed by the sum of the scores of weight loss, stool consistency and rectal bleeding. Maximum score: 12.
